# Author’s Response to Peer Reviews of “Health Care System Overstretch and In-Hospital Mortality of Intubated Patients With COVID-19 in Greece From September 2020 to April 2022: Updated Retrospective Cohort Study”

**DOI:** 10.2196/59637

**Published:** 2024-06-10

**Authors:** Theodore Lytras

**Affiliations:** 1 School of Medicine, European University Cyprus, Engomi, Cyprus

**Keywords:** COVID-19, pandemic, health care disparities, intensive care unit, ICU, right to health, quality of care, intubation, mortality, health disparity, health inequality, surveillance data, in-patient, mortality, COVID-19 patient, hospitalization, disparity, inequality, surveillance, health care system, Greece, region, Delta, Omicron, vaccination, vaccine, public health, patient load, deterioration, time

*This is the authors’ response to peer-review reports for “Health Care System Overstretch and In-Hospital Mortality of Intubated Patients With COVID-19 in Greece From September 2020 to April 2022: Updated Retrospective Cohort Study.”*

## Round 1 Review

### Anonymous [1]

*“We aimed to update this analysis [2] to include the large “Delta” and “Omicron” waves that affected Greece during 2021‐2022.”*

*So why did you analyze data also from 2020?*

Response: Because we needed to consider the entirety of the relevant data (in the context of our aim to describe the relationship between patient load and in-hospital mortality), and not only the new data of 2021 and 2022. Looking at the totality of the evidence is good practice, as we are sure you would agree.

*“Vaccination did not affect the mortality of these already severely ill patients.”*

*Did you check how many months before they had received the vaccine? How many of the participants were not vaccinated?*

Response: Examining the time lag between vaccination and disease onset was not part of our analysis; 11,944 of 14,011 patients (85.2%) were unvaccinated, as is already reported on Table 1; we have now included this information in the text and in the abstract.

*“Vaccination did not show a statistically significant association with mortality, regardless of the number of doses received (Figure 2).”*

*This needs rephrasing if all participants were vaccinated at least once.*

Response: No, they were not. In fact, most participants (85.2%) were unvaccinated. The described lack of association refers to our multivariable Poisson model (summarized in Figure 2), where you can see that the hazard ratios for 1, 2, or 3 doses versus zero doses are all statistically nonsignificant. Having said that, this finding is not surprising: the COVID-19 vaccine is very effective in preventing severe disease and thus death, but *once severe disease does occur*, surviving it depends on the health care received, not on vaccination.

*“Between 1 September 2020 and 3 April 2022...”*

*I see that your previous study included data up to May 2021. Thus, there is a lot of overlap in the data of these two studies.*

Response: Our current study is clearly described as being an update of the previous study (which we cite as reference 3). That study analyzed 6282 patients, whereas the current one analyzes 14,011 patients, more than twice as many. Also the original only covered the Alpha variant wave, whereas the current study extends that to the Delta and Omicron waves. It is therefore a meaningful and important update, not at all salami-sliced as you may imply.

*There are many errors in the use of English and typos which makes it difficult to understand. See examples below:*

*“Mortality was significantly higher above 400 patients”*

*This is not very clear (please rephrase).*

*“...with an adjusted Hazard Ratio of 1.22, 95% CI: 1.09‐1.38)”*

*Where does the parenthesis open?*

*“...rising progressively up to 1.48 (95% CI: 1.31‐1.69) for 800+ patients.”*

*This is not clear.*

*“...we found no statistically association between mortality and...”*

*Please correct typo.*

Response: Thank you for your comment; we have rephrased/corrected all of the above.

*It would be good to include DOIs to all references.*

Response: Thank you, we now follow the JMIR citation style (as included in Zotero), which includes PMIDs to all relevant references.

### Reviewer CF [3]

*Abstract has to clarify the goal, sample, results, and health and social implications to cope with the next pandemics to improve health care.*

Response: Thank you for your comment. We have extensively amended the abstract. At the same time, we are reluctant to overinterpret our findings (with respect to “social implications”) especially in the context of the abstract. Still, we already make a clear reference to that in our conclusion section of the abstract (“This highlights the need for urgent strengthening of health care services in Greece, ensuring equitable and high-quality care for all”).

*Introduction is poor and has to better clarify the research questions of this study and provide more theoretical background about strategies of prevention and good governance to cope with the pandemic crisis. After that they can focus on the topics of this study to provide a correct analysis for fruitful discussion (see suggested readings that must be all read and used in the text).*

Response: Thank you, but the aim of our study is not about general “strategies of prevention and good governance.” It is specifically (and narrowly) about illustrating the association between patient load during the pandemic and in-hospital mortality of severely ill COVID-19 patients. To reflect that, our introduction is short and to-the-point, stating the context and aims of the study and setting up our methods, results and discussion.

*Methods of this study are not clear. The section of Materials and Methods must be restructured with the following 3 points in the same order...*

Response: Thank you for your comment. We have already structured the methods with these 3 items in the same order, just not using subheadings. The first paragraph includes the sample, data source, and variables, and the second paragraph describes the statistical analysis.

*Results: It is not clear why authors apply a Mann-Whitney test, considering the large sample it is better to apply an independent sample t test, or some other parametric test. This additional test can support better results if they are reliable.*

Response: With all due respect, this is incorrect. The nonparametric Mann-Whitney is used in Table 1 to compare the age distribution between patients who died and those who survived, because age does not conform to a normal distribution. It is not a matter of sample size. In any event, the point is moot, since the difference is so large that both the Mann-Whitney and 2-sample *t* test produce *P*<.001.

*Table 1: avoid acronyms; specify ICU as intensive care unit. Same comments for Figure 2.*

Response: We have defined the acronym ICU at first use (both in the Abstract, and in the Methods section of the manuscript) as is standard practice.

*Discussion: First, authors have to synthesize the main results in a simple table to be clear for readers and then show what this study adds compared to other studies. In addition, authors should discuss the type of mechanical ventilation, because studies show that invasive ventilation (intubation) creates VAP (ventilator-associated pneumonia), and a lot of people intubated for COVID-19 died from this problem rather than COVID-19. Countries that have reduced mortality, such as Germany and New Zealand, used mainly noninvasive ventilation, which can better treat patients and avoid mortality with new technology.*

Response: Thank you, but we have already summarized all the main results in Figure 2 (which includes all effect sizes and 95% CIs). All our patients were mechanically ventilated; therefore, we do not have empirical data to discuss the point that the reviewer suggests. Thus, if we were to do so, it would be pure speculation.

*Conclusion has to be added as an autonomous section. Conclusion not to be a summary, but authors have to focus on manifold limitations of this study and provide suggestions for health, crisis management, and social policy, as well as how nations can prevent, with good governance and new technology in artificial ventilation, the next pandemics and improve health care with vaccination, noninvasive ventilation, and nonpharmaceutical measures of control. In this manner the paper can provide useful policy implications for Greece and improve health care for the next pandemics.*

Response: Thank you, but this is a short communication and—more importantly—we want to avoid overinterpreting our data or venturing beyond what our data can support (which is what the reviewer suggests). May we also point out that our original analysis (of which this current study is an update) became hotly debated in Greece, which is another reason we wish to be cautious in our interpretation. Nevertheless, the very last sentence of our Discussion section clearly and unambiguously offers our conclusion and policy implications: “The findings highlight the need for urgent strengthening of health care services in Greece in order to improve their performance and ensure equitable access to high-quality care for all.”
